# Cuticular hydrocarbons are associated with mating success and insecticide resistance in malaria vectors

**DOI:** 10.1038/s42003-021-02434-1

**Published:** 2021-07-26

**Authors:** Kelsey L. Adams, Simon P. Sawadogo, Charles Nignan, Abdoulaye Niang, Douglas G. Paton, W. Robert Shaw, Adam South, Jennifer Wang, Maurice A. Itoe, Kristine Werling, Roch K. Dabiré, Abdoulaye Diabaté, Flaminia Catteruccia

**Affiliations:** 1 Department of Immunology and Infectious Diseases, Harvard T.H. Chan School of Public Health, Boston, MA USA; 2 Department of Medical Biology and Public Health, Institut de Recherche en Science de la Santé (IRSS), Bobo-Dioulasso, Burkina Faso; 3 Cummings School of Veterinary Medicine, Tufts University, North Grafton, MA USA; 4 Harvard Center for Mass Spectrometry, Harvard University, Cambridge, MA USA; 5 Department of Entomology, Pennsylvania State University, State College, PA USA

**Keywords:** Entomology, Behavioural ecology, Sexual selection

## Abstract

*Anopheles coluzzii* females, important malaria vectors in Africa, mate only once in their lifetime. Mating occurs in aerial swarms with a high male-to-female ratio, where traits underlying male mating success are largely unknown. Here, we investigated whether cuticular hydrocarbons (CHCs) influence mating success in natural mating swarms in Burkina Faso. As insecticides are widely used in this area for malaria control, we also determined whether CHCs affect insecticide resistance levels. We find that mated males have higher CHC abundance than unmated controls, suggesting CHCs could be determinants of mating success. Additionally, mated males have higher insecticide resistance under pyrethroid challenge, and we show a link between resistance intensity and CHC abundance. Taken together, our results suggest that CHC abundance may be subject to sexual selection in addition to selection by insecticide pressure. This has implications for insecticide resistance management, as these traits may be sustained in the population due to their benefits in mating even in the absence of insecticides.

## Abstract (web summary)

In this study, Adams et al. investigate the effect of cuticular hydrocarbons on mating success in natural mosquito mating swarms. These hydrocarbons confer both higher mating success and increased resistance to pyrethroid, suggesting sexual selection for insecticide resistance in this population secondary to mating success.

## Introduction

Two of the major malaria vectors in sub-Saharan Africa, *Anopheles gambiae* and *Anopheles coluzzii* of the *A. gambiae* complex, are largely monandrous, which means the lifetime reproductive fitness of females depends on a single mating event^1^. In these anopheline species, mating occurs in aerial swarms where males heavily outnumber females. This type of skewed sex ratio can be associated with female mate choice and/or scramble mating competition among males^2,3^. Swarm initiation remains poorly understood, but it is thought that these mosquitoes integrate visual signals from geographic markers and lighting, circadian cues, acoustic signals, and volatile pheromones to identify the presence of conspecific individuals of the opposite sex^4–7^. In spite of substantial research efforts, close-range cues involved in mate choice remain largely unknown in *Anopheles*. Harmonic convergence, the adjustment of wingbeat frequencies between a male and female, is observed leading up to close-range interactions in *Anopheles* like in other mosquito species, but there is no evidence that it increases the likelihood of successful copulation in *A. gambiae*^8–10^. Other studies have striven to understand whether male fitness, reflected by body size, determines mating outcomes, with unclear and conflicting conclusions^5,11–13^. When a swarming male approaches a female, there is substantial contact between their legs and abdomens after which, in successful mating events, the male grasps the female and completes copulation^14^. During these close-range interactions, females can exhibit rejection behavior before copulation starts^4,15,16^.

Contact pheromones, including cuticular hydrocarbons (CHCs), are widely used by insects during social or sexual communication^17,18^. CHCs are waxy molecules derived from fatty acids via a biosynthetic process that involves desaturases, elongases, fatty acid synthases, and cytochrome P450 enzymes^19,20^. Biosynthesis occurs in specific cells called oenocytes, from where they are transported to the surface of the cuticle by lipophorin proteins, where they can regulate permeability in addition to playing pheromonal roles^21^. These compounds are chemically diverse, and are thought to be highly tuned to environmental pressures such as aridity as well as subject to sexual selection, resulting in plasticity in their composition and levels^22^.

In mosquitoes, the role of CHCs in communication has not been fully elucidated, although reports indicate that stripping the cuticle with solvent^23–25^ or treating virgin females with CHC extracts from either males or females^26^ can reduce insemination rates, suggesting that CHCs may alter mate recognition or selection. Further, recent data in *Anopheles stephensi* mosquitoes shows that males treated with the CHC heptacosane inseminate more females compared to untreated controls^7^, indicating a potential role for CHCs in mating behavior.

The possibility of sexual selection for CHCs in *Anopheles* is particularly interesting because it is already known that these traits are selected for by insecticide pressure. Cuticular insecticide resistance (IR), a thickening of the cuticle caused by increased deposition of CHCs, cuticular proteins, and chitin, leads to reduced or slowed insecticide penetrance^27–29^. There have now been several observations of higher CHC abundance in resistant mosquito populations, and this has been linked to overexpression of two cytochrome P450 enzymes, *CYP4G16* and *CYP4G17*, that act as decarbonylases in the last steps in the CHC biosynthesis pathway^27,28^. Therefore, if CHCs are implicated in female mate choice or male competition during swarming, cuticular thickening due to selective pressures imposed by insecticides may also affect male mating success. Understanding whether CHCs affect both mating biology and IR is particularly relevant in areas of Africa where widespread IR is threatening the efficacy of our best malaria control tools, which are predominantly based on the use of insecticides against vector species.

Here we investigated whether CHCs are associated with male mating success and with IR in field *A. coluzzii* populations from Burkina Faso. We show that males that successfully mate with females in natural mating swarms have higher total abundance of CHCs, and that these males survive longer during insecticide exposure. Moreover, we identify signatures of cuticular resistance in these populations and show their association with survival after insecticide exposure. Our data support a model by which CHCs play overlapping roles in male mating success and IR, suggesting higher CHC abundance may be under selection by sexual selection as well as by insecticide pressure. These findings have important repercussions for the spread of IR as well as for currently proposed genetic control strategies in *Anopheles*.

## Results

### Mated males have higher total CHC abundances in natural *A. coluzzii* swarms

To investigate whether CHC levels affect male mating success we decided to study males from natural mating swarms. Colonization of mosquitoes to confined laboratory conditions can affect mating behavior and select for traits that are not necessarily relevant in field conditions^30^. To avoid these effects, we collected mated and unmated control mosquitoes from natural *A. coluzzii* swarms in VK7, a village in the Vallée du Kou area near Bobo Dioulasso, Burkina Faso. Mated males were collected *in copula*, while the unmated groups were collected at different time points during the swarming period (throughout peak swarming, when mating activity is high, or at late time points after mating activity has tapered off) in random sweeps. We therefore refer to these males as unmated controls, which are likely a reflection of the average swarming male.

**Fig. 1:**
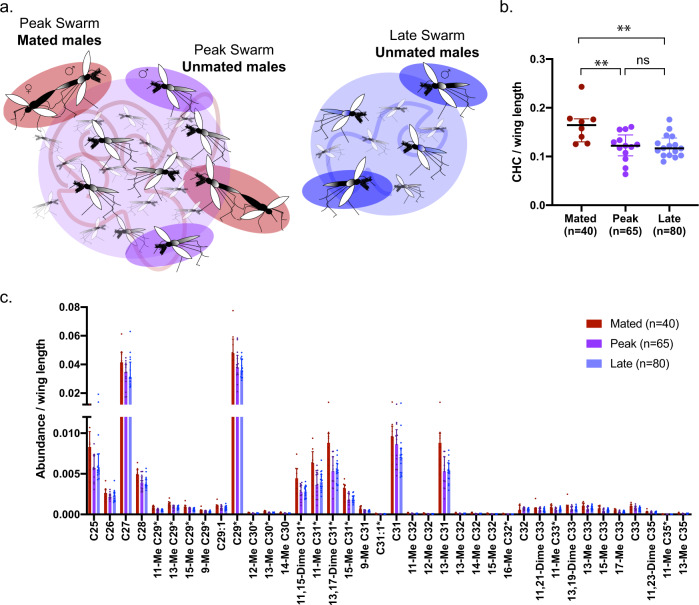
Successful *A. coluzzii* males in natural swarms have higher abundance of CHCs. **a** Scheme of captures of male groups from mating swarms, showing mated males (left image, red), unmated males from the peak swarm (left image, purple), and unmated males collected late in the swarm (right image, blue). **b** Total abundance of CHCs is higher in mated compared to unmated males when captured at either peak or late time points during the swarm (Tukey’s multiple comparisons, *p* = 0.0045 (mated vs peak unmated), *p* = 0.0041 (mated vs late unmated)). The mean sum of response ratios for all CHCs divided by the mean wing length for each sample is shown. Error bars represent SD, and *n* describes total number of mosquitoes. **c** Mated males have higher abundance of 15 of 38 CHCs compared to both unmated males captured at the peak or late time point detected by GC–MS, shown here as the median response ratio to a pentadecane internal standard and normalized to wing length. Nomenclature for each compound indicates position of methyl (Me) or Dimethyl (Dime) groups on the carbon chain. Error bars represent interquartile ranges; Benjamini–Hochberg corrected *p* values from Mann–Whitney tests are displayed in full in Supplementary Table 1. Asterisks are indicated next to names of compounds with statistically significant differences in both peak and late unmated groups compared to the mated group.

We extracted CHCs from multiple pools of five males from either mated or unmated groups (Fig. 1a), and extracts were submitted for gas chromatography mass spectrometry (GC–MS) analysis to retrieve quantitative and qualitative information of the CHC profiles. While all groups of males showed the same diversity of 38 CHC compounds regardless of mated status (Supplementary Table 1), mated males captured *in copula* had higher (by 1.37-fold) levels of CHCs compared to unmated controls from either peak or late time points after normalizing for wing length, a proxy for adult size (Fig. 1b). No difference was instead detected between peak and late control groups (Fig. 1b). When the two unmated groups were pooled, the greater CHC abundance of mated males was maintained (Supplementary Fig. 1a). Wing length was not significantly different between mated and unmated groups in all comparisons (Supplementary Fig. 1b, c). Among the 38 identified compounds, 15 had increased abundance in mated males compared to both peak and late unmated control groups (Fig. 1c). The representation of each individual compound relative to the total abundance (proportional abundance) was similar between the three groups, indicating that the major differences between CHCs of these groups are quantitative and not qualitative (Supplementary Fig. 1d).

### Mated males from natural mating swarms survive longer under permethrin exposure

**Fig. 2:**
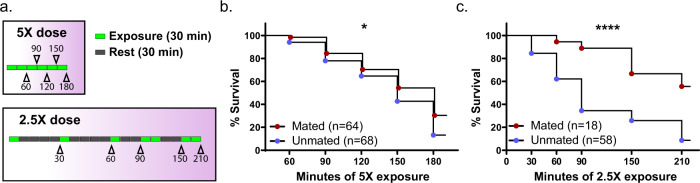
Successful males have higher permethrin resistance. Mated and control unmated males were captured from natural swarms as in Fig. 1a. **a** These males were exposed to either a continuous 5× dose of permethrin (upper panel) or a series of 30 or 60 min 2.5× permethrin exposures (lower panel) and monitored for knockdown after each exposure. Schematics represent 30-min time intervals as either exposure periods (green) or rest periods (gray). Arrowheads denote time points for survival monitoring, labeled according to the cumulative minutes of permethrin exposure. Mated males survived longer on average to these permethrin exposures compared to unmated males for both the **b** 5× (*p* = 0.036) and **c** 2.5× (*p* < 0.0001) doses (log-rank tests). *n* represents total number of mosquitoes.

Based on these findings, and evidence that CHCs are linked with IR^27^, we next directly investigated whether mated males also have higher resistance to permethrin, an insecticide widely used on Long Lasting Insecticidal Nets (LLINs) in this area^31^. We collected mated and unmated (from the late time point) males from natural swarms as above (Fig. 1a). Given that no difference appears between CHCs of peak versus late unmated controls we compared only the late group in the remainder of the study. Our reasoning is that the late time point is the most relevant control as by then no more females join the swarm and therefore males in this group are highly unlikely to mate that same evening. The day following collection we exposed these two groups to a 3.75% (5×) continuous dose of permethrin using WHO bioassay cylinders and permethrin-impregnated papers^32^. Mosquitoes were monitored for knockdown (failure to fly) every 30 min, and their total time to knockdown (referred to here as “survival”) was recorded (Fig. 2a upper panel). When exposed to this dose of permethrin, mated males were 1.65 times more likely to survive the exposure (log-rank test, *p* = 0.036), with a median survival 30 min longer than unmated controls (Fig. 2b).

To further resolve the differences in survival between these mosquitoes, we next utilized an intermittent exposure regime using a 1.875% (2.5×) dose of permethrin, and also included recovery periods between exposures (Fig. 2a lower panel). When exposed to this dose, mated males were again more likely to survive (by 3.72 times) compared to unmated controls (log-rank test, *p* < 0.0001) (Fig. 2c). While control males had a median time to death of 90 min, in the mated group more than half of the males were still alive after 210 min of exposure, so their median time to death was undefined. We again observed no significant difference in wing length of mated compared to unmated males (Supplementary Fig. 2). When combined, our results show that males that are successful at mating exhibit greater resistance to insecticides.

### *A. coluzzii* populations from Vallée du Kou show evidence of cuticular resistance

**Fig. 3:**
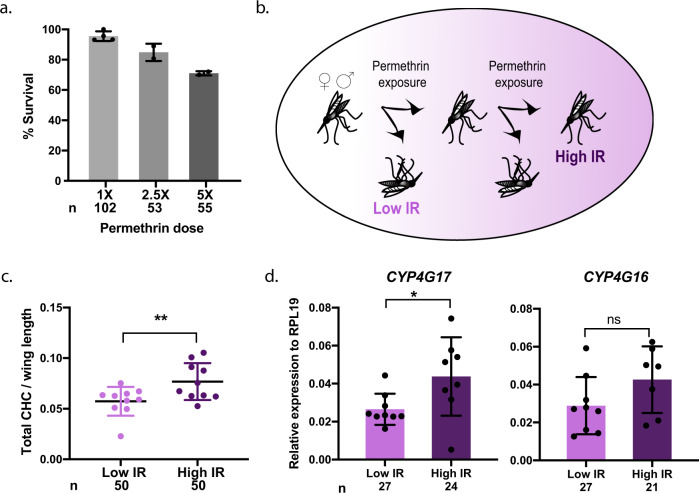
CHC levels are correlated with insecticide resistance intensity in field-derived *A. coluzzii*. **a** Insecticide resistance bioassays using 1×, 2.5×, or 5× permethrin-impregnated papers show high insecticide resistance in *A. coluzzii* adults collected as larvae from VK5 breeding sites. **b** Scheme of experimental design showing how mosquitoes from larval collections were categorized as Low IR or High IR based on their survival to two sequential permethrin exposures. **c** High IR mosquitoes show higher abundance of CHCs compared to Low IR mosquitoes (Generalized Linear Model, *p* = 0.0083) after normalizing for wing length and accounting for sex within the model (*p* = 0.2651). Mean and SD are shown. **d** High IR females also show higher transcript abundance of *CYP4G17* (unpaired *t*-test, *p* = 0.0346), but not *CYP4G16* (unpaired *t*-test, *p* > 0.05) by qRT-PCR. Bars represent mean and SD; *n* represents total number of mosquitoes.

The evidence of a relationship between CHC abundance, mating success, and resistance to pyrethroids prompted us to determine whether cuticular resistance is a mechanism acting in these mosquito populations, as suggested by other studies^28,33–35^. We first confirmed high intensity of pyrethroid resistance in this region by exposing adult *A. coluzzii* mosquitoes to permethrin, after collecting them as larvae from natural breeding sites in the Vallée du Kou. These tests detected nearly 100% survival at the standard 0.75% (1×) permethrin dose, and >70% survival at both 2.5× and 5× doses (Fig. 3a), in agreement with previous reports^31^.

We next tested whether CHC abundance was correlated with IR intensity in the same population using age-matched *A. coluzzii* mosquitoes collected from larval breeding sites. We first separated mosquitoes according to their IR intensity (Low or High IR) based on their survival to two consecutive permethrin exposures. We classified as Low IR mosquitoes those who died after a single exposure to permethrin, while High IR mosquitoes were those who also survived a second exposure (Fig. 3b). Permethrin doses used were different in males and females, as females survived longer when exposed to the same doses (Supplementary Fig. 3a). Data from males and females were pooled to increase sample size as we saw no difference in total CHC abundances between sexes (Supplementary Fig. 3b), though some qualitative differences were observed (Supplementary Table 2). We detected a 1.33-fold increase in the total abundance of CHCs in High IR mosquitoes compared to the Low IR groups from the same population (Generalized Linear Model, *p* = 0.0083) (Fig. 3c). High IR mosquitoes were larger **(**Supplementary Fig. 3c), but we accounted for size differences by normalizing CHCs to wing length (Fig. 3c).

To gain further evidence of cuticular resistance in those populations, we also investigated transcript levels for the cuticular resistance genes *CYP4G16* and *CYP4G17* among High and Low IR females. Sample collection and quality were not sufficient for data collection from males. *CYP4G17* expression was significantly higher in the High IR group (unpaired *t*-test, *p* = 0.0346), while differences in *CYP4G16* expression trended in the same direction but were not significant (Fig. 3d). All together, these data provide evidence for cuticular resistance in this mosquito population, confirming published studies^28,33^, and show a direct association between CHC abundance and IR intensity in age-matched mosquitoes.

## Discussion

In *Anopheles* mosquitoes, the traits defining male attractiveness and competitiveness during mating are not well understood. Here we show that males that are successful in mating swarms have a higher abundance of CHCs, which could act as contact pheromones during interactions with females. Our data indicate that though relative abundances of different CHCs are similar among *A. coluzzii* males, greater total abundance is associated with mating success (Fig. 1b). While CHC quantity could reflect increased male fitness involved in male–male competition, we speculate that it more plausibly reflects increased attractiveness to females as CHCs are commonly used as contact pheromones in other insects^18^, and because of documented female rejection behavior during close-range interactions that supports female choice upon contact with males^4,15,16^. Recent findings that show *A. stephensi* males treated with heptacosane (an abundant CHC also detected in our analyses) have higher insemination rates compared to control treated males^7^. This also suggests that CHCs render males more attractive or at least more recognizable to females, as it is unlikely that coating males with exogenous CHC would increase male–male competitiveness. Mating competition experiments that quantify rates of mate acceptance or rejection using males coated with different quantities of CHC would more conclusively demonstrate whether females choose to mate with males with higher CHCs. *A. coluzzii* females may only assess the abundance of one or a few important compounds when evaluating male fitness, but there is precedent for association of total CHC abundance with mating outcomes. In *Drosophila serrata*, for example, overall CHC levels increase under conditions of sexual selection^36^, while in *Gnatocerus* flour beetles and *Cyphoderris* sagebrush crickets, total abundance is thought to play a role in mate choice, though CHC composition is also involved^36–38^.

Notably, the upregulation of the CHC biosynthetic pathway has advantages beyond male mating success. These compounds form a waxy seal on the exterior epicuticle that regulates permeability to water, insecticides, and other chemicals, and may therefore provide benefits in conditions of environmental stress^22,39^. Indeed, in a region of Burkina Faso where high pressure from LLINs and indoor residual spraying have caused the emergence and spread of a number of IR mechanisms, including cuticular thickening^27,28^, we found that males that were successful in mating were more resistant to pyrethroid insecticides (Fig. 2). Consistent with previous studies, we also detected the upregulation of an important marker (*CYP4G17*) of cuticular resistance^27^ in mosquitoes that are more resistant to pyrethroids (Fig. 3d). Combined, this evidence points to the possibility that CHC abundance may not only be selected for by insecticide pressure, but also propagated by the increased mating success of individuals that possess cuticular resistance traits, unveiling an unexpected link between sexual selection and the failure of our best malaria control tools. Although most insecticide-based interventions are mainly targeted at adult females, adult males also rest indoors^40^ besides being exposed to insecticides during larval development, so increased CHC abundance is likely to be directly beneficial by increasing both the likelihood that males will survive long enough to mate and their chances of being successful during swarming events.

Our finding that CHCs act as dual traits in *A. coluzzii*, with roles in both mating behavior and in withstanding environmental pressures, is consistent with reports in *Drosophila* where hydrocarbons involved in mate choice are thought to have evolved differentially in different species based on environmental conditions like aridity of their ecological niches^19^. Increased CHC production is likely to impose fitness costs that can only be offset in specific environmental conditions such as high insecticide usage or aridity, and indeed there is evidence that CHCs also contribute to desiccation tolerance in *A. gambiae*^41–43^. Fitness costs of CHC production have been reported in *Drosophila*, where there appears to be a trade-off between CHC abundance and oogenesis, to the point that in the absence of sexual selection total CHC content is reduced in both male and female flies^36,44^. In the Vallée du Kou area where we conducted our studies, in addition to heavy insecticide usage mosquitoes are exposed to other significant environmental factors like a dry season that subjects mosquitoes to desiccation stress. Both these conditions are likely to promote increased CHC production even when a balance must be struck between abundance of these cuticular pheromones and their presumed fitness costs.

Our data could be explained by different evolutionary models. In a “good genes” model, high levels of CHCs would increase offspring fitness in environments with high insecticide usage or desiccation stress and would therefore be honest indicators of male fitness^45^. Under a “sexy son” model or Fisherian process instead, one might expect to see cuticular resistance in all populations of *A. coluzzii* due to runaway sexual selection^46,47^. Neither model can be excluded given there is no data on the heritability of CHC levels, and the occurrence of cuticular resistance has not been systematically tested largely due to the lack of simple and reliable molecular diagnostic tools. If males with high CHC abundance are more successful even in the absence of insecticide or desiccation pressures, the sexy son hypothesis^47^ (or a sensory exploitation model^48^) may better explain these observations. Other studies exploring the validity of the good genes hypothesis have compared the adaptive capacity of insect colonies in the presence of sexual selection, or when insects are deprived of mate selection^49^. Forced mating techniques are possible in *A. gambiae*^50^ and similar experiments where sexual selection is removed could be performed. If sexual selection accelerates insect adaptation to a novel stress (such as routine insecticide exposure), this would be evidence for a good genes model.

It is important to note that additional IR mechanisms exist in this mosquito population including metabolic and target site resistance^33,34^. We cannot exclude that they contributed to the survival differences in our experiments, nor do we argue that cuticular resistance is necessarily equally or more dominant as a mechanism. However, the fact that we detected CHC differences between mosquitoes with low and high IR (Fig. 3c), despite the potential contributions of other mechanisms, suggests cuticular resistance is important. In future studies, it will be critical to explore how different ecological factors shape mating success and sexual selection in *Anopheles* vectors from other malaria-endemic regions.

Using wild-caught mosquitoes from natural swarms in our study has some inevitable limitations. We could not prove that control males had not mated during that evening, but we expect this to be the case for two reasons: (1) returning to the swarm on the same evening is unlikely, given the steep energy demands associated with copulation^11^, and (2) the highly biased sex ratios and large numbers of males in these swarms (several thousands) make sampling of returned males improbable. We also assume that mating does not change the CHC profile of males, an assumption supported by previous evidence^26^. Also, due to the lack of appropriate high-resolution age-grading technology, we cannot control for the age of males caught from natural swarms. Although we cannot rule out age as a confounding factor entirely, this parameter is not likely to critically influence our results due to the following arguments. First, it has been shown that age is not associated with mating in natural swarms^12^. Moreover, Sawadogo et al. also showed that 80–90% of *A. gambiae* swarming males are >4 days old and insemination rates are highest using males between 4 and 8 days of age^12^. Based on estimates of daily survival, as few as 5% of males may live longer than 8 days^51^, and we therefore expect that the vast majority of our cohort of swarming males are between 4 and 8 days old. Second, IR does wane with age, but in the range of 4–8 days of age these effects are subtle, at least in females^52^. Lastly, though the impact of age on the total CHC abundance in males is not fully understood, a previous study observed an increase in CHCs with age in *A. gambiae* females^43^. Together these studies show that effects of age on CHC abundance and IR may act in opposing directions, making it highly unlikely that age explains our findings here. Importantly, when age was controlled by using mosquitoes from larval collections from breeding sites in the same region, we still observed increased CHC abundance in mosquitoes that were more resistant to permethrin (Fig. 3b).

Finally, our findings that males with reduced CHC levels are less competitive in mating swarms also have repercussions for vector control strategies currently in the design stage that propose to release sterile or genetically modified males for malaria control. Given our results, laboratory-derived males lacking IR mechanisms such as cuticular resistance would likely be less successful in certain environmental settings when mating in swarms alongside wild males, whether at the level of female mate choice or male competition. This reinforces the need to take into account the local genetic background and the presence of IR mechanisms before releasing laboratory-reared males in vector control programs.

## Materials and methods

### Mating captures from natural swarms

Using small nets, mating couples of *A. coluzzii*, a subset of which were genotyped using primers from Santolamazza et al.^53^, were manually caught from swarms in Vallée du Kou village 7 by trained personnel^54,55^. Samples collected for analysis of CHCs in swarming males (Fig. 1) were obtained during September 2017, while samples collected in September 2018 were used to determine IR intensities in swarming males (Fig. 2). During collections, the nets were verified to contain one male and one female prior to being mouth-aspirated from the net into a small cup covered with netting. Nets that contained more than one male were discarded. For the unmated control groups, males were collected by one or several sweeps of a net through the swarm between 3 and 15 min into the swarm for the peak swarm time point, or 17–20 min into the swarm for the late swarm time point. Males from these sweeps were aspirated from nets into cups. Mosquitoes were given cotton soaked with 10% sugar solution and transported from field sites to an insectary in a vehicle. There they were additionally given cotton soaked in water overnight. CHCs were collected from these males 24 h later.

### GC–MS samples

For all GC–MS samples, pools of five mosquitoes were submerged in 200 µL hexane for 30 min. Hexane was evaporated and samples were stored at room temperature. Just prior to GC–MS, samples were resuspended in 200 µL hexane containing pentadecane as an internal standard of known quantity (1.53 µg/sample). Three microliters sample was injected into an Agilent fused-silica capillary column of cross-linked DB-5MS (30 m × 0.25 mm × 0.25 µm). The GC conditions were as follows: inlet and transfer line temperatures, 290 °C; oven temperature program, 50 °C for 0.6 min, 50 °C/min to 80 °C for 2 min, 30 °C/min to 120 °C, 5 °C/min to 310 °C for 20 min, 50 °C/min to 325 °C for 10 min; inlet helium carrier gas flow rate, 1 mL/min; split mode, splitless. These conditions are optimized for detection and resolution of lower chain length molecules.

### GC–MS analysis

A CHC accurate-mass target database was built based on the retention time comparison method and on the characterized ions reported by Caputo et al. to identify the compounds from the GC–MS run^56^. The relative response of the peak area of the extracted ion chromatogram of a target relative to the pentadecane internal standard was used to generate a quantitative value for each compound, called the response ratio.

### Absolute abundance

We compared this value between samples to look at the differences in absolute quantity of each CHC, after accounting for wing length of the mosquitoes in each sample. The sum of response ratios for all CHC compounds identified in a sample was used to compare total abundance of CHCs. We only included in our analyses compounds that were identified in at least 80% of all samples for each run. Data from one day of sample collection from swarms were excluded because all samples failed to detect eight compounds that were detected in samples from all other collection days, and also expressed less than 25% of the total CHC abundance compared to the average. Statistics were performed as follows: for mated versus unmated comparisons, data were checked for normality prior to running one-way ANOVA with Tukey’s multiple comparisons for mated versus peak unmated versus late unmated comparisons. For Low IR versus High IR comparisons: data were checked for normality prior to running a Generalized Linear Model for Low IR versus High IR comparisons accounting for sex within the model. For mosquitoes binned according to IR intensity, animals in the Low IR groups tended to have fewer legs remaining post-exposure to permethrin, so the number of legs was normalized by removing the appropriate number of legs from High IR mosquitoes prior to hexane extraction to mirror the Low IR group.

### Proportional abundance

We calculated and compared the relative abundance of each compound in all samples to determine whether the proportional representation of each compound was different between groups. To compare abundance of individual compounds, data were not distributed normally for all compounds so Mann–Whitney tests were used to determine statistical differences between groups, and a Benjamini–Hochberg correction for multiple comparisons was subsequently performed with *Q* = 0.2.

### Wing length measurement

Wings were imaged and measured from the proximal wing notch to the distal tip of the third cross vein using ImageJ^57,58^. All measurements were taken by the same person for consistency. The response ratio (relative to pentadecane standard) of total CHCs was divided by wing length (mm) to give normalized values. After testing to verify that the data fall into a normal distribution, wing lengths were compared using unpaired *t*-tests or one-way ANOVA with Tukey’s multiple comparisons in GraphPad Prism 8.4.3 (GraphPad Software Inc. USA).

### IR bioassays

Permethrin-impregnated papers were prepared at 0.75% (1×), 1.875% (2.5×), or 3.75% (5×) permethrin (Sigma-Aldrich PESTANAL^®^ analytical standard) concentrations, weight/volume. Standard WHO bioassay tubes were used to expose mosquitoes to permethrin-impregnated papers for a given period. For standard WHO bioassays^32^, mosquitoes were exposed to permethrin for 1 h, and 24 h recovery time was allowed before assessing mortality. For non-standard time-to-death assays, mosquitoes were exposed for either 30- or 60-min periods and monitored for survival after a given recovery time, as shown in Fig. 2a. To compare survival duration, log-rank tests were performed in JMP Pro 13 (SAS Corp. US). For non-standard assays binning mosquitoes as Low IR or High IR mosquitoes were exposed to permethrin in two subsequent intervals as follows: for females, 60 min 5× permethrin followed by 120 min rest, followed by 30 min 5× permethrin and another 120 min rest (Supplementary Fig. 3a, upper panel). For males: 30 min 2.5× permethrin exposure followed by 120 min rest, 30 min 2.5× permethrin, 120 min rest (Supplementary Fig. 3a lower panel.). Survival was assessed after each rest period. Pilot experiments were used to determine permethrin doses that yielded approximately equal numbers of High and Low IR mosquitoes.

### Transcriptional analysis of IR genes

After IR bioassays were performed and categorized females as Low IR or High IR, whole bodies were placed in RNA*later*™ (Thermo Fisher Scientific) in pools of three. A 2-h post-exposure time point was used to reduce degradation of mosquitoes that had died during exposure, while allowing some recovery time for those that were only knocked down. After 24 h at 4 °C, excess RNA*later* was removed, and samples were frozen at −20 °C. Samples were later homogenized in 600 µL TRI reagent^®^ (Thermo Fisher Scientific). RNA was then extracted according to modified manufacturer instructions, including three washes of the RNA pellet with 75% ethanol. Samples were treated with TURBO™ DNase (Thermo Fisher Scientific) prior to quantification with a NanoDrop Spectrophotometer 2000c (Thermo Fisher Scientific). At this stage, we determined that male samples yielded poor quantity and quality RNA, while female samples were adequate for cDNA synthesis and qRT-PCR. Briefly, cDNA synthesis was performed using ~2 µg RNA per sample in a reaction volume of 100 µL. Published primer sequences for *CYP4G16* and *CYP4G17* were obtained as previously described^27^. qRT-PCR reactions were run on a StepOnePlus thermocycler using SYBR-Green Master Mix (Thermo Fisher Scientific), with 300 nM primers and 1:3 dilutions of cDNA in a 15 µL reaction. *RPL19* was used as a house-keeping gene to normalize Ct values obtained for each sample. Expression values were then compared using unpaired *t*-tests using GraphPad Prism after verifying that data were distributed normally.

### Mosquito larval collections and rearing

Larval breeding sites in Vallée du Kou village 5 were surveilled daily during September 2018, and larvae were collected between the L3 and pupal stage. Predators and competing mosquito species were removed, and mosquitoes were reared from these stages in natural spring water with TetraMin^®^ powder (Tetra) fed daily. Mosquitoes were sex-separated as pupae under a light microscope and given ad libitum access to cotton soaked with water and a 10% sugar solution throughout adulthood with a 12 h light 12 h dark cycle.

### Statistics and reproducibility

Detailed statistical methods are described within the relevant methods sections. CHC data were collected from swarming males on three separate nights. Night of collections, and swarm that males were collected from were determined to be nonsignificant factors on CHC abundance. Survival of swarming males to insecticide exposure was evaluated from collections on six separate nights. Exposures were performed with mating status blinded, for five replicates for 5× survival and 1 replicate for 2.5× survival due to limitations of sample collection. CHC data were collected from Low and High IR from only one of three experiments, and RNA was collected from females from three replicates.

### Reporting summary

Further information on research design is available in the Nature Research Reporting Summary linked to this article.

## Supplementary information

Description of Supplementary Files

Supplementary Information

Supplementary Data 1

Reporting Summary

## Data Availability

All pertinent data are available within the manuscript or upon request. GC–MS data, wing lengths, survival proportions, and transcriptional RNA data are available within Supplementary Data 1.
