# Supplementary material for: Cuticular hydrocarbons are associated with mating success and insecticide resistance in malaria vectors
Source: Commun Biol. 2021 Jul 26;4:911. doi: 10.1038/s42003-021-02434-1 (PMC8313523; doi:10.1038/s42003-021-02434-1)
Supplement: Supplementary file 1 — Description of Supplementary Files [file 42003_2021_2434_MOESM1_ESM.pdf]

Description of Additional Supplementary Files

File name: Supplementary Data 1

Description: The four tabs in this data file include: Raw CHC profile data, wing length data, male survival to permethrin and qRT-PCR data.
