# Supplementary material for: Cuticular hydrocarbons are associated with mating success and insecticide resistance in malaria vectors
Source: Commun Biol. 2021 Jul 26;4:911. doi: 10.1038/s42003-021-02434-1 (PMC8313523; doi:10.1038/s42003-021-02434-1)
Supplement: Supplementary file 2 — Supplementary Information [file 42003_2021_2434_MOESM2_ESM.pdf]

Supplementary information for:

Cuticular hydrocarbons are associated with mating success and insecticide resistance in malaria vectors

Kelsey L. Adams[1], Simon P. Sawadogo[2], Charles Nignan[2], Abdoulaye Niang[2], Douglas G. Paton[1], W. Robert Shaw[1], Adam South[1,3], Jennifer Wang[4], Maurice A. Itoe[1], Kristine Werling[1,5], Roch K. Dabiré[2], Abdoulaye Diabaté[2], Flaminia Catteruccia[1*]

[1]Department of Immunology and Infectious Diseases, Harvard T.H. Chan School of Public Health, Boston, MA 02115

[2]Department of Medical Biology and Public Health, Institut de Recherche en Science de la Santé (IRSS), Bobo-Dioulasso, Burkina Faso, BP 545

[3]Cummings School of Veterinary Medicine, Tufts University, North Grafton, MA, 01536

[4]Harvard Center for Mass Spectrometry, Harvard University, Cambridge, MA, 02138

[5]Department of Entomology, Pennsylvania State University, State College, PA 16802

Corresponding author: Dr. Flaminia Catteruccia, Building 1 Rm. 103, 665 Huntington Ave, Boston 02115, 617-432-1773, fcatter@hsph.harvard.edu

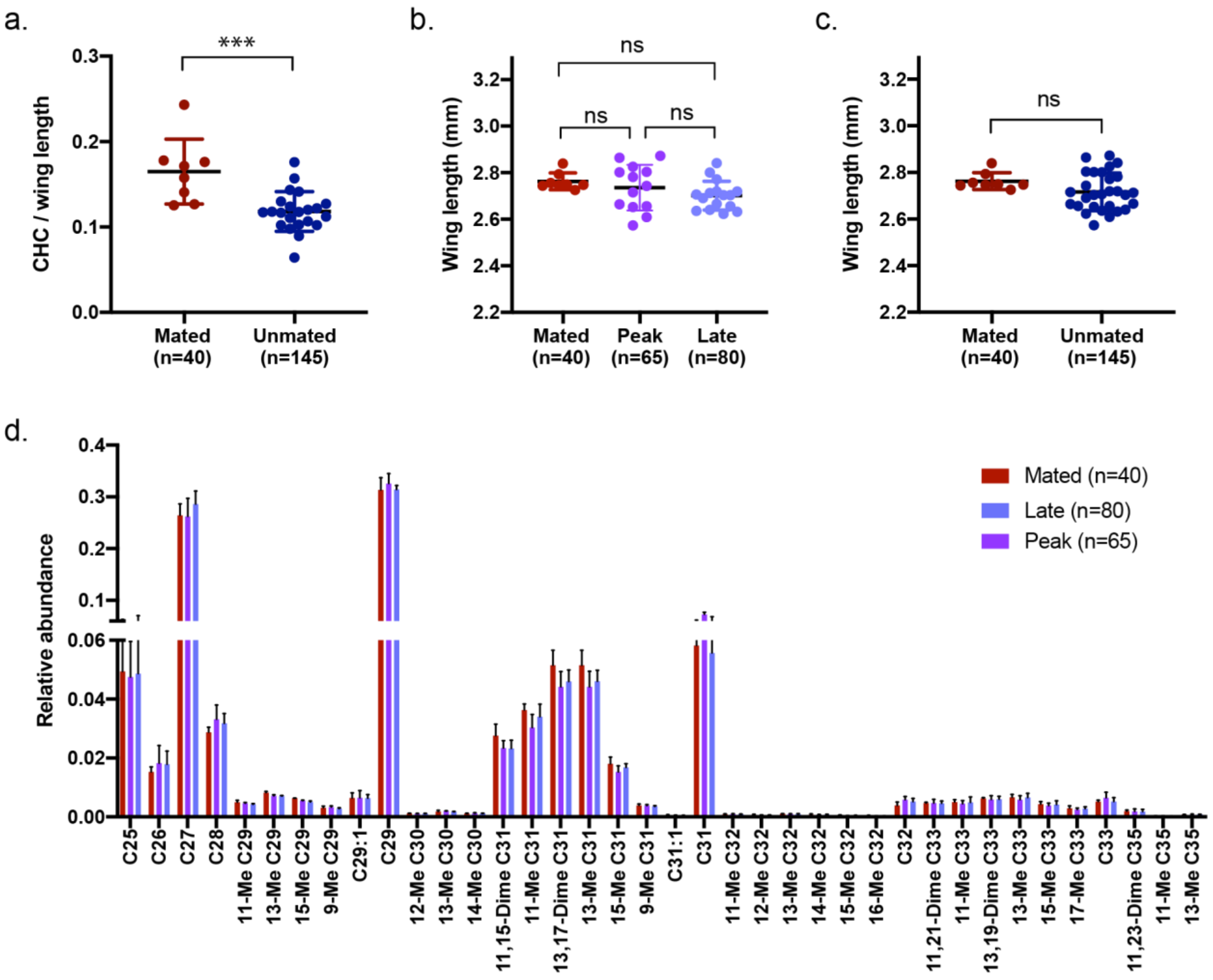

Supplementary Figure 1: **Neither body size nor proportional abundances of CHCs are different between mated and control groups.** (a) CHC abundance is higher in mated males compared to all unmated groups when peak and late time points are combined (Un-paired t-test, *p*=0.0003). Mean and SD are represented. (b) Wing lengths are not different between either late or peak unmated males compare to mated males (Tukey's multiple comparisons, *p*>0.05 for all comparisons). The mean wing length of each pool of 5 males is represented as each data point. Mean and SD are shown. (c) Wing lengths are not different between mated males and unmated males when peak and late time points are combined (Un-paired t-test, *p*>0.05). The mean wing length of each pool of 5 males is represented by each data point. Mean and SD are shown. (d)

The proportional representation of all CHCs is not different between mated and unmated individuals from the peak or late time point of the swarm (Mann-Whitney tests, $p>0.05$, medians with interquartile ranges represented). *n* describes total number of mosquitoes.

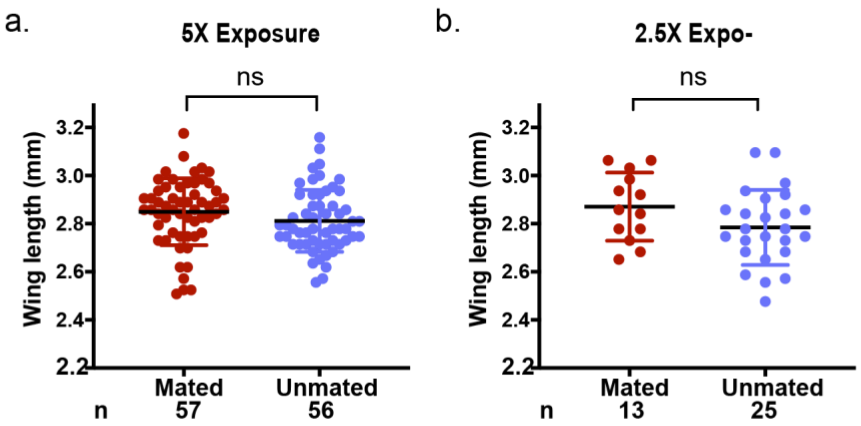

Supplementary Figure 2: Wing lengths of mated and unmated males challenged with permethrin. Wing lengths of individual males exposed to permethrin in (a) 5X continuous exposure experiment or (b) 2.5X intermittent exposure experiment do not show significant differences (unpaired t-tests, $p>0.05$, bars represent mean and SD). n represents total number of mosquitoes.

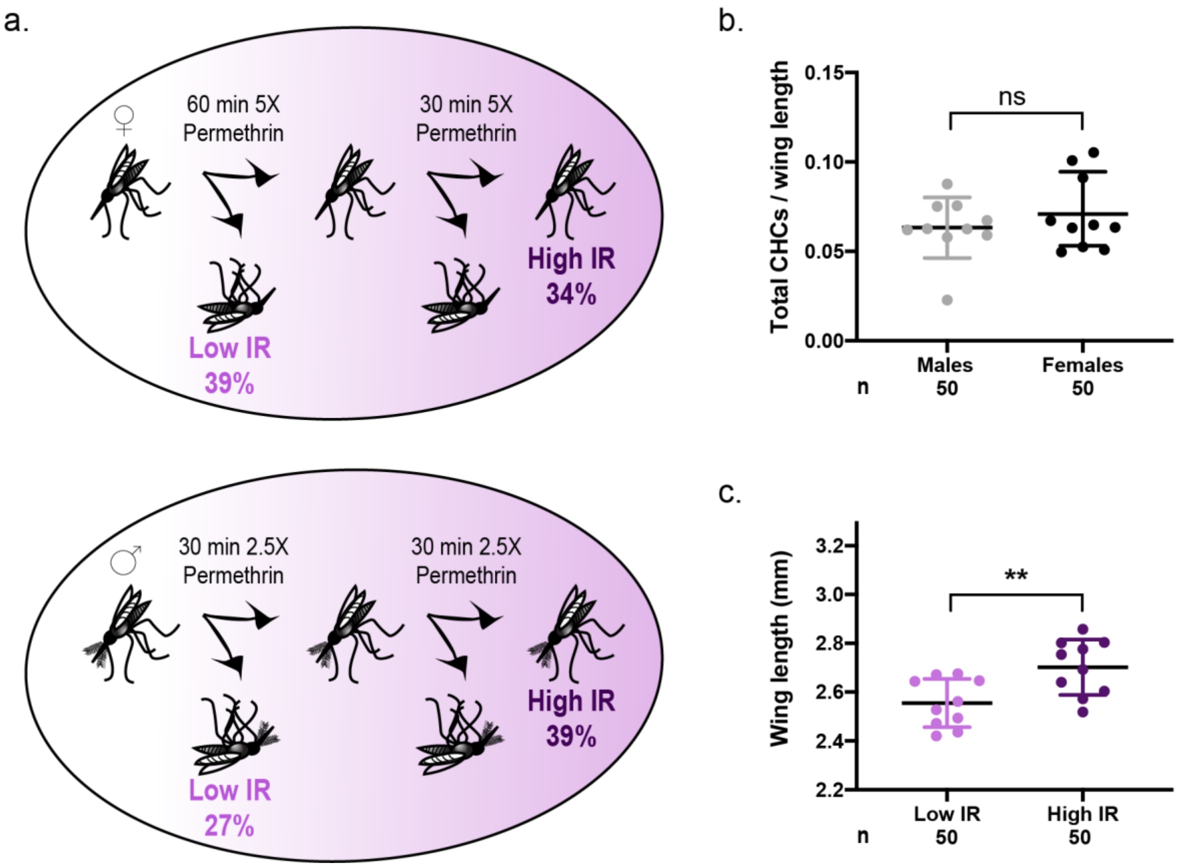

Supplementary Figure 3: Wing lengths and CHC abundances of Low and High IR mosquitoes (a) Schematics of procedure to separate females (i) and males (ii) into Low and High IR resistance groups based on their survival to two consecutive exposures to permethrin, with percentages showing the proportion of the population falling into either Low IR (died after the first exposure) or High IR (survived both exposures). (b) Total CHC abundances are the same in males compared to females, normalized to wing length (unpaired t-test, $p>0.05$, bars represent mean and SD. (c) Average wing lengths of each pool of 5 mosquitoes submitted for GC-MS are greater in High IR compared to Low IR groups (unpaired t-test, $p=0.0066$, bars represent mean and SD). *n* represents total number of mosquitoes.

Supplementary Table 1: **Absolute abundances of CHCs in mated and unmated *An. coluzzii* males from natural swarms.** Values show response ratios normalized to wing length. *p* values are calculated by Mann-Whitney tests, and then corrected for multiple comparisons using Benjamini-Hochberg procedure. Significant corrected *p* values ($p<0.05$) are shown in bold.

	**Abundance**			***P* value (Mann-Whitney)**		***P* value (Benjamini-Hochberg corrected)**	
Compound	Mated	Peak unmated	Late unmated	Mated vs Peak	Mated vs Late	Mated vs Peak	Mated vs Late
C25	0.008303	0.005654	0.005585	0.0535	0.1924	0.075	0.09210526
C26	0.002572	0.002135	0.002292	0.2381	0.3196	0.09342105	0.09473684
C27	0.041469	0.034294	0.030597	0.0446	0.0523	0.06578947	0.07105263
C28	0.00488	0.003899	0.003631	0.0638	0.0192	0.07894737	**0.04605263**
**11-Me C29**	0.000791	0.000572	0.000509	0.0077	0.0017	**0.02236842**	**0.00789474**
**13-Me C29**	0.001233	0.000853	0.000791	0.0077	0.0013	**0.025**	**0.00526316**
**15-Me C29**	0.000884	0.000681	0.000589	0.0082	0.0045	**0.03026316**	**0.01710526**
**9-Me C29**	0.000492	0.000397	0.000317	0.0093	0.0126	**0.03289474**	**0.03684211**
C29:1	0.001053	0.000759	0.00073	0.0465	0.0382	0.06973684	0.06315789
**C29**	0.047758	0.038032	0.03596	0.0159	0.0009	**0.04210526**	**0.00263158**
**12-Me C30**	0.000217	0.000137	0.000136	0.0012	0.0002	**0.00394737**	**0.00131579**
**13-Me C30**	0.000343	0.000225	0.000209	0.0168	0.0034	**0.04342105**	**0.01184211**
14-Me C30	0.000188	0.000146	0.000127	0.0446	0.0071	0.06447368	**0.01973684**
11-Me C31	0.004451	0.002746	0.002847	0.0246	0.0808	0.05131579	0.08289474
**11,15-Dime C31**	0.006308	0.003572	0.00399	0.0126	0.0087	**0.03552632**	**0.03157895**
**13-Me C31**	0.008805	0.005304	0.005263	0.0077	0.0036	**0.02631579**	**0.01447368**
**13,17-Dime C31**	0.003175	0.001739	0.001848	0.0077	0.0036	**0.02368421**	**0.01315789**
**15-Me C31**	0.000662	0.000461	0.000409	0.012	0.0057	**0.03421053**	**0.01842105**
9-Me C31	8.04E-05	6.06E-05	5.74E-05	0.0535	0.0126	0.07368421	**0.03815789**
**C31:1**	0.009543	0.008639	0.006976	0.0077	0.0071	**0.02894737**	**0.02105263**
C31	0.000146	0.000108	0.000102	0.4137	0.023	0.09605263	0.05
11-Me C32	0.000114	6.53E-05	6.45E-05	0.1213	0.0284	0.08552632	0.05657895
12-Me C32	0.008805	0.005306	0.005264	0.1042	0.0629	0.08421053	0.07763158
13-Me C32	0.000165	0.000107	0.000106	0.0368	0.0382	0.05921053	0.06184211
14-Me C32	0.000135	8.15E-05	0.000089	0.0134	0.068	**0.03947368**	0.08026316
15-Me C32	0.000102	4.49E-05	4.93E-05	0.0246	0.0192	0.05263158	**0.04473684**
**16-Me C32**	7.42E-05	2.94E-05	2.31E-05	0.0045	0.0016	**0.01578947**	**0.00657895**
C32	0.000551	0.000676	0.0006	0.6452	0.9761	0.09868421	0.1
**11-Me C33**	0.000776	0.000505	0.00051	0.0159	0.023	**0.04078947**	**0.04868421**
11,21-Dime C33	0.000851	0.000484	0.000549	0.1614	0.0382	0.08947368	0.06052632
13-Me C33	0.000948	0.000605	0.00066	0.0199	0.0702	**0.04736842**	0.08157895
13,19-Dime C33	0.001077	0.000629	0.000726	0.1257	0.0448	0.08815789	0.06710526
15-Me C33	0.000734	0.000403	0.000479	0.0246	0.0448	0.05394737	0.06842105
17-Me C33	0.000502	0.000237	0.0003	0.0077	0.0263	**0.02763158**	0.05526316
C33	0.000939	0.000758	0.000616	0.5466	0.0523	0.09736842	0.07236842
**11-Me C35**	0.000314	0.000234	0.000222	0.0018	0.0028	**0.00921053**	**0.01052632**
11,23-Dime C35	4.56E-05	1.77E-05	2.19E-05	0.1614	0.1234	0.09078947	0.08684211
13-Me C35	0.000143	7.85E-05	8.75E-05	0.0288	0.0607	0.05789474	0.07631579

Supplementary Table 2: Abundances of CHCs in Low IR or High IR mosquitoes. Values show response ratios normalized to wing length.

Compound	Abundance Low IR males ($x10^{-3}$)	Abundance High IR males ($x10^{-3}$)	Abundance Low IR females ($x10^{-3}$)	Abundance High IR females ($x10^{-3}$)
13-Me C29	0.29624	0.40962	0.18132	0.25524
15-Me C29	0.22167	0.30402	0.16447	0.20917
11-Me C31	1.22651	1.54715	0.78088	1.3426
13-Me C31	1.49892	2.20471	0.79387	1.24947
15-Me C31	0.54675	0.78977	0.38437	0.58238
11-Me C33	0	0	0.30465	0.41216
13-Me C33	0.48426	0.42648	0.29724	0.38535
13,19-Dime C33	0	0	0.35104	0.33374
15-Me C33	0.28042	0.37498	0.15118	0.21748
13-Me C35	0	0	0.12137	0.17807
C27:1	5.7665	7.06931	5.9586	8.27392
C29:1	4.4635	5.49036	4.63286	6.84128
C17	0	0	0.084602	0.085738
C29	15.78802	19.82221	19.84549	27.57241
C31	4.76514	5.85578	3.60508	4.62783
