# Supplementary material for: Cuticular hydrocarbons are associated with mating success and insecticide resistance in malaria vectors
Source: Commun Biol. 2021 Jul 26;4:911. doi: 10.1038/s42003-021-02434-1 (PMC8313523; doi:10.1038/s42003-021-02434-1)
Supplement: Supplementary file 4 — Reporting Summary [file 42003_2021_2434_MOESM4_ESM.pdf]

nature research

Corresponding author(s): Flaminia Catteruccia
Last updated by author(s): Jun 21, 2021

Reporting Summary

Nature Research wishes to improve the reproducibility of the work that we publish. This form provides structure for consistency and transparency in reporting. For further information on Nature Research policies, see our Editorial Policies and the Editorial Policy Checklist.

Statistics

For all statistical analyses, confirm that the following items are present in the figure legend, table legend, main text, or Methods section.

n/a	Confirmed	
☐	☒	The exact sample size (*n*) for each experimental group/condition, given as a discrete number and unit of measurement
☐	☒	A statement on whether measurements were taken from distinct samples or whether the same sample was measured repeatedly
☐	☒	The statistical test(s) used AND whether they are one- or two-sided<br>*Only common tests should be described solely by name; describe more complex techniques in the Methods section.*
☐	☒	A description of all covariates tested
☐	☒	A description of any assumptions or corrections, such as tests of normality and adjustment for multiple comparisons
☐	☒	A full description of the statistical parameters including central tendency (e.g. means) or other basic estimates (e.g. regression coefficient) AND variation (e.g. standard deviation) or associated estimates of uncertainty (e.g. confidence intervals)
☐	☒	For null hypothesis testing, the test statistic (e.g. *F*, *t*, *r*) with confidence intervals, effect sizes, degrees of freedom and *P* value noted<br>*Give P values as exact values whenever suitable.*
☒	☐	For Bayesian analysis, information on the choice of priors and Markov chain Monte Carlo settings
☒	☐	For hierarchical and complex designs, identification of the appropriate level for tests and full reporting of outcomes
☒	☐	Estimates of effect sizes (e.g. Cohen's *d*, Pearson's *r*), indicating how they were calculated

Our web collection on statistics for biologists contains articles on many of the points above.

Software and code

Policy information about availability of computer code

Data collection	NA
Data analysis	NA

For manuscripts utilizing custom algorithms or software that are central to the research but not yet described in published literature, software must be made available to editors and reviewers. We strongly encourage code deposition in a community repository (e.g. GitHub). See the Nature Research guidelines for submitting code & software for further information.

Data

Policy information about availability of data

All manuscripts must include a data availability statement. This statement should provide the following information, where applicable:

- Accession codes, unique identifiers, or web links for publicly available datasets
- A list of figures that have associated raw data
- A description of any restrictions on data availability

Raw data from figures 1-3 are available as supplementary information or supplementary data.

Field-specific reporting

Life sciences study design

All studies must disclose on these points even when the disclosure is negative.

Sample size	We are limited by numbers of mated mosquitoes that can be captured. We obtained as many mating couples as possible from 5 or more visits to field sites on each of two sequential trips to Burkina Faso, and used data from those where meaningful numbers of mated mosquitoes were obtained.
Data exclusions	Mass spectrometry data were excluded from samples collected from one swarm visit because the data yielded no detection of some compounds that were present in all other samples, and total detection of CHCs was less than 25% of samples collected on other nights.
Replication	Each of 3 nights of collection and up to 3 swarms per night replicated the findings in Figure 1. Data from findings in figure 2B were replicated 5 times, although data collection in figure 2C and figure 3C were performed only once due to time, budget and sample collection constraints.
Randomization	Although randomization of field mosquitoes was not possible due to their inherent status of mated or unmated upon collection, covariates such as swarm, date of collection, and size were all determined to be non-significant covariates by multivariate testing.
Blinding	Data was blinded for mass spectrometry analysis, as well as for insecticide exposure survival analysis (mated and unmated groups were unknown to the researcher while scoring knockdown).

Reporting for specific materials, systems and methods

We require information from authors about some types of materials, experimental systems and methods used in many studies. Here, indicate whether each material, system or method listed is relevant to your study. If you are not sure if a list item applies to your research, read the appropriate section before selecting a response.

Materials & experimental systems

n/a	Involved in the study	
☒	☐	Antibodies
☒	☐	Eukaryotic cell lines
☒	☐	Palaeontology and archaeology
☐	☒	Animals and other organisms
☒	☐	Human research participants
☒	☐	Clinical data
☒	☐	Dual use research of concern

Methods

n/a	Involved in the study	
☒	☐	ChIP-seq
☒	☐	Flow cytometry
☒	☐	MRI-based neuroimaging

Animals and other organisms

Policy information about studies involving animals; ARRIVE guidelines recommended for reporting animal research

Laboratory animals	This study did not involve laboratory animals
Wild animals	Field-caught An. coluzzii (species verified by PCR (Santolamazzaet al, 2008)) males and females were used in this study. They were either captured as adults from mating swarms and transported back to the lab by vehicle in paper cups covered by netting and cotton, or were collected as larvae from natural breeding sites, and transported by vehicle to the lab. Mosquitoes were eventually killed by either freezing or insecticide exposure.
Field-collected samples	CHC samples were collected in hexane and stored at room temperature until analyzed. RNA samples were collected in RNA later, and stored at -20 degrees C until extraction, after which they were stored at -80 degrees C.
Ethics oversight	No ethical approval was required

Note that full information on the approval of the study protocol must also be provided in the manuscript.
